# Supplementary material for: The Effect of Vitamin E Supplementation on Serum Aminotransferases in Non-Alcoholic Fatty Liver Disease (NAFLD): A Systematic Review and Meta-Analysis
Source: Nutrients. 2023 Aug 25;15(17):3733. doi: 10.3390/nu15173733 (PMC10490270; doi:10.3390/nu15173733)
Supplement: Supplementary file 1 [file nutrients-15-03733-s001.zip › nutrients-2546194-supplementary.pdf]

Table S1. Summary Risk of bias assessment of RCTs using the ROB-2 tool.

	D1	D2	D3	D4	D5	Overall
Adamo, 2013	X	+	+	-	+	X
Anushiravani, 2019	+	+	+	+	-	-
Basu, 2014	+	+	+	+	+	+
Bril, 2019	+	+	+	+	+	+
Dufour, 2006	-	+	-	-	-	X
Ekhlasi, 2016	-	+	+	+	+	-
Ghergherehchi, 2013	-	+	+	+	-	-
Lavine, 2011	+	+	+	+	+	+
Magosso, 2013	+	+	-	+	+	-
Pervez, 2020	-	+	+	+	-	-
Sanyal, 2010	+	+	+	+	+	+
Wang, 2008	X	+	+	+	+	X

Domains

D1: bias arising from the randomization process
D2: bias due to deviations from intended interventions
D3: bias due to missing outcome data
D4: bias in measurement of the outcome
D5: bias in selection of the reported result

Judgement

X High
- Some concerns
+ Low

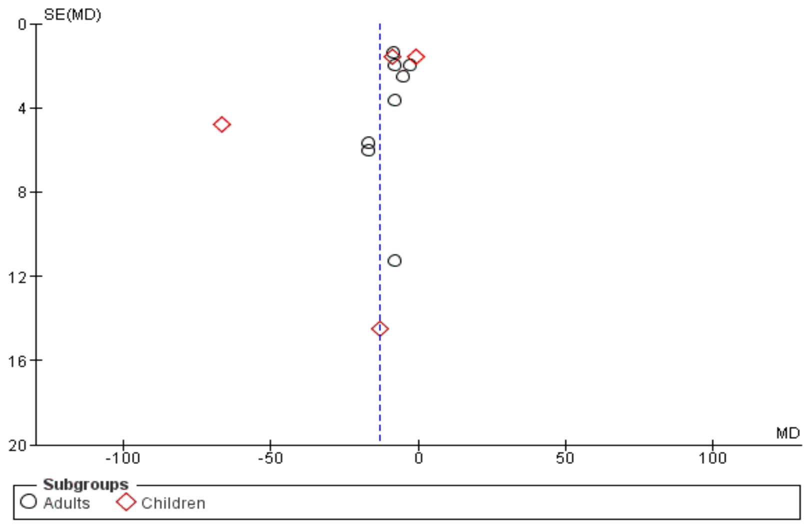

Figure S1. Funnel plots for publication bias regarding vitamin E supplementation and mean differences (MD) in ALT levels among adults and children.

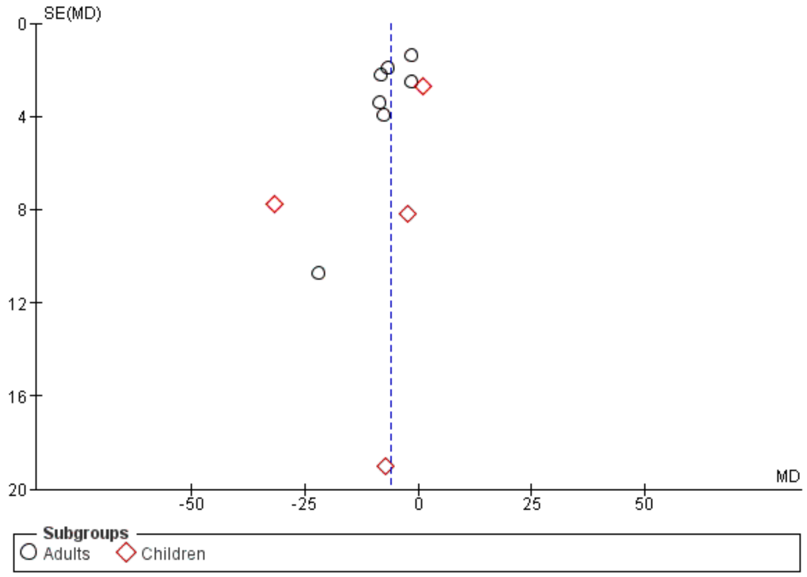

Figure S2. Funnel plots for publication bias regarding vitamin E supplementation and mean differences (MD) in AST levels among adults and children.
